# Feasibility and safety of a self-developed sleeve for the endoscopic removal of refractory foreign body incarceration

**DOI:** 10.3389/fsurg.2023.1150004

**Published:** 2023-05-03

**Authors:** Guangqiu Yu, Li Li, Yirui Zhang, Xiaohuan Zhong, Jing Wang, Ling Jiang, Duanmin Hu, Weixia Zhou

**Affiliations:** ^1^ Department of Gastroenterology, The Second Affiliated Hospital of Soochow University, Suzhou, China; ^2^ Department of Gastroenterology, The Third People's Hospital of Dalian, Dalian, China

**Keywords:** self-developed sleeve, refractory foreign body incarceration, endoscopy, upper gastrointestinal tract (UGIT), esophagus

## Abstract

**Objective:**

This study aimed to assess the feasibility and safety of a novel self-designed sleeve for the endoscopic removal of a refractory incarcerated foreign body in the upper gastrointestinal tract (UGIT).

**Methods:**

An interventional study was conducted between June and December 2022. A total of 60 patients who underwent an endoscopic removal of a refractory incarcerated foreign body from the UGIT were randomly allocated to the self-developed sleeve and the conventional transparent cap. The study evaluated and compared the operation time, successful removal rate, new injury length at the entrance of the esophagus, new injury length at the impaction site, visual field clarity, and postoperative complications between the two groups.

**Results:**

The success rates of the two cohorts in the foreign body removal display no significant discrepancy (100% vs. 93%, *P* = 0.529). Nevertheless, the methodology of the novel overtube-assisted endoscopic foreign body removal has culminated in a significant reduction in the removal duration [40 (10, 50) min vs. 80 (10, 90) min, *P* = 0.01], reduction in esophageal entrance traumas [0 (0, 0) mm vs. 4.0 (0, 6) mm, *P* < 0.001], mitigation of injuries at the location of the foreign body incarceration [0 (0, 2) mm vs. 6.0 (3, 8) mm, *P* < 0.001], an enhanced visual field (*P* < 0.001), and a decrement in postoperative mucosal bleeding (23% vs. 67%, *P* < 0.001). The self-developed sleeve effectively negated the advantages of incarceration exclusion during removal.

**Conclusion:**

The study findings support the feasibility and safety of the self-developed sleeve for the endoscopic removal of a refractory incarcerated foreign body in the UGIT, with advantages over the conventional transparent cap.

## 1. Introduction

The presence of a foreign body in the upper gastrointestinal tract (UGIT) is a frequent digestive tract emergency encountered in clinical settings. The majority of patients commonly present with new-onset chest pain, odynophagia, or the feeling of an esophageal foreign body sensation after meals (1). However, it can also result in complications such as perforation, bleeding, and other adverse effects (2, 3).

Researchers have reported an increased risk of laceration when the foreign body has a sharp edge and is similar in length to the esophageal inner diameter, which is approximately 22–26 mm (4). An esophageal foreign body firmly embedded in the wall is more likely to cause mucosal damage during the embedding and removal procedures. Improper or delayed treatment of refractory foreign body incarceration can lead to severe complications such as mediastinitis, aortoesophageal fistula, pneumothorax, and pericardial effusion (5–8).

The outer sleeve is one of the most frequently implemented endoscopic tools for diagnosis and treatment, with its primary function being to safeguard the mucosa and reduce any associated damage (9). The outer sleeve currently implemented in clinical settings has an inner diameter of 14–21 mm, making it challenging to maneuver and extract larger-sized foreign bodies. The identification of the optimal method for the safe and effective removal of large and sharp foreign bodies firmly embedded in the gastrointestinal tract is a challenging aspect of emergency endoscopic treatment.

The tip of our independently developed endoscopic sleeve has been found to facilitate the removal of foreign body impactions, with an inner diameter of 18 mm and an outer diameter of 19 mm. The remotely inclined plane diameter measures 25 mm, which corresponds with the width that the esophagus can accommodate, allowing for the retrieval of all foreign bodies present within the esophagus.

In this study, we evaluated the efficiency and safety of a novel endoscopic foreign body removal sleeve (the self-developed sleeve) independently designed by our team to facilitate the acute management of refractory foreign body incarceration in the UGIT. To achieve this, we analyzed the clinical data of patients who underwent a soft gastroscopic removal of refractory foreign body incarceration in the UGIT at our hospital.

## 2. Materials and methods

### 2.1. Study design

This was an observational interventional study. We examined the difference between different auxiliary methods (the self-developed sleeve vs. transparent cap group) in terms of the operation time, removal success rate, new injury length at esophagus entrance, new injury length at foreign body entrapment, visual field clarity, and postoperative complications. The self-developed sleeve used in this research is a patent invented by the author, with patent number ZL 2020 2 2283346.0.

### 2.2. Patients

Patients who underwent an endoscopic treatment in our hospital due to refractory foreign body incarceration in the UGIT between June and December 2022 were observed.

(1) Inclusion criteria: adult patients with UGIT foreign bodies discovered by computed tomography (CT) and treated endoscopically; CT-estimated foreign body length ≥ 15 mm; foreign body embedded in the esophageal wall observed endoscopically; and postoperative follow-up of clinical symptoms *via* laboratory examination or CT showing whether there were complications.
(2) Exclusion criteria: patient age <18 years; CT-confirmed perforation of the digestive tract; or incomplete follow-up data.

Prior to participation, all patients underwent informed consent procedures. Approval for this study was obtained from the ethics committee of The Second Affiliated Hospital of Soochow University (no. jd-lk-2021-068-01), and the study was registered with the China Clinical Trial Registration Center (no. ChiCTR2200063289).

### 2.3. Equipment and procedures

Every patient underwent a preoperative CT scan to identify the exact location of the foreign object and its relation to surrounding organs. Prior to the procedure, the patients received either local anesthesia with 2% lidocaine and sedation or general anesthesia with propofol administered *via* the pharynx. A highly experienced gastroenterologist conducted the examination using an Olympus GIF-HQ290/H260 endoscope system (Olympus Optical Corporation, Tokyo, Japan) with a transparent cap of 12.4 mm inner diameter (D-201-11804; Olympus Optical Corporation), in accordance with the highest professional standards.

**Figure 1:**
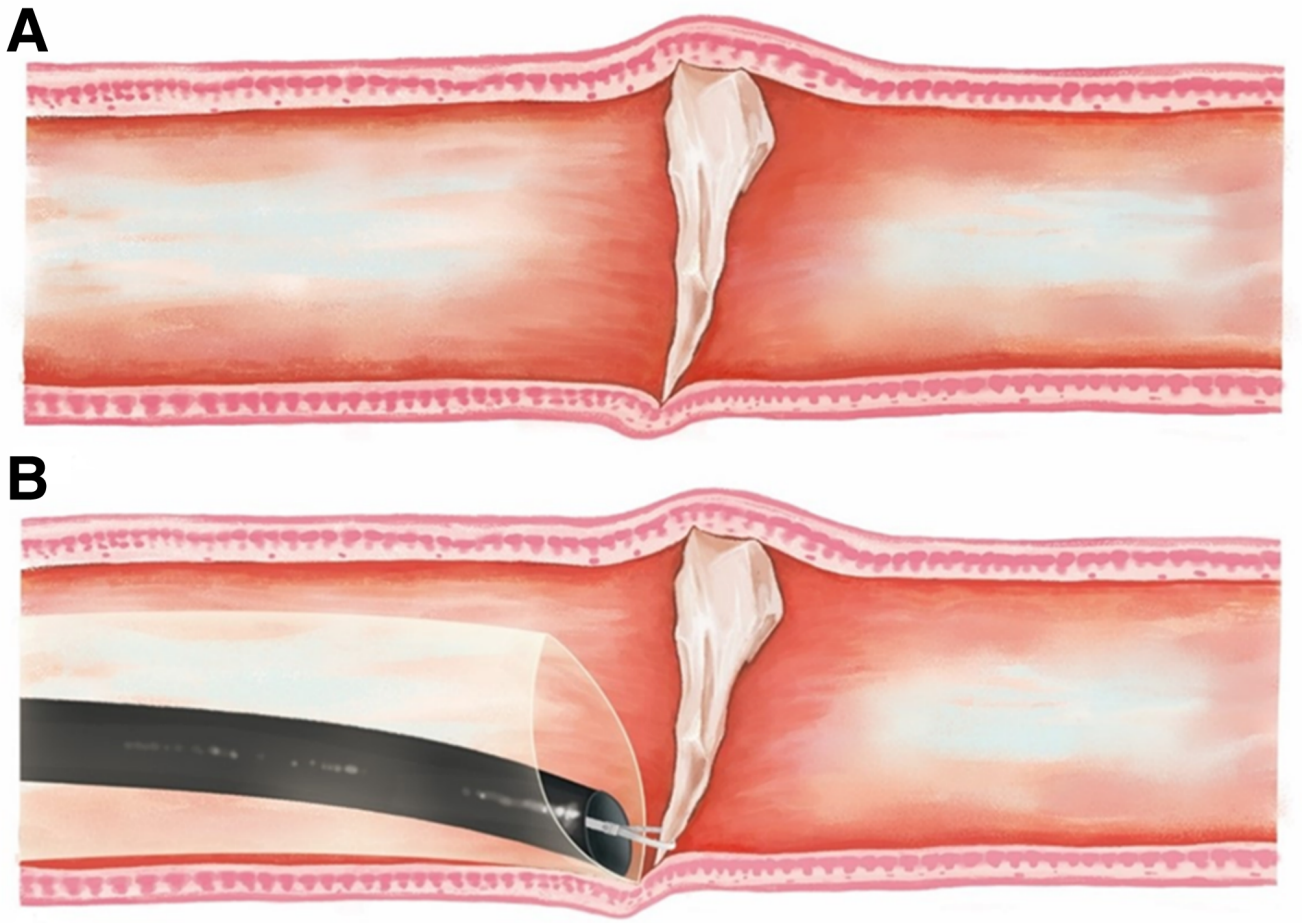
A foreign object impaction (**A**), and the extraction of the foreign object using the novel sheath (**B**). During the extraction, the distal tip of the sheath was employed to apply pressure on the esophageal wall with the lens body, affording the capability to precisely guide the tip of the sheath toward the foreign body. Once the target was in view, the foreign object was efficiently extracted using rat tooth forceps as the auxiliary instrument.

The design of the self-developed sleeve incorporated a soft, lubricated outer sleeve (with an inner diameter of 18 mm, outer diameter of 19 mm, distal slope length of 25 mm, tube wall thickness of 1.0 mm, and length options of 18, 28, or 50 cm) inserted into the gastroscope to reach the foreign object under gastroscopic visualization. The distal tip of the sleeve, equipped with a lens body, exerts pressure on the esophageal tube wall, allowing it to approach the deeply embedded tip of the foreign object. The foreign object is then removed with the aid of an auxiliary instrument rat tooth forceps. Figure 1 is a schematic diagram.

When the foreign object can be fully retracted into the outer sleeve, it will be removed with the gastroscope, while the sleeve remains in place to initiate hemostasis. The transparent tube wall facilitates observation of the extent of the damage before the outer sleeve is safely removed. In cases where the maximum transverse diameter of the foreign object is over 2.5 cm (excluding its length), the tip is drawn into the cannula before removal, and the foreign body is then pulled out with the gastroscope. Subsequently, the scope is re-inserted to evaluate a potential mucosal damage.

### 2.4. Data collection

Two experts in digestive endoscopy evaluated all study data. In cases of discrepancies, a third endoscopy expert was consulted for a final evaluation.

Data, including each patient's clinical information, foreign object location (cervical, thoracic, or abdominal esophageal segment), foreign object size, operation duration (from endoscope insertion to foreign object removal), the success rate of removal, and the length of additional mucosal damage at the entrance of the esophagus and at the site of foreign object embedding (using rat tooth forceps), were assessed.

Visual field definition was classified as follows: grade A referred to the observation of the foreign object shape, position, and esophageal mucosa; grade B referred to the observation of the foreign object shape, position, and some esophageal mucosa; and grade C indicated the nonobservance of the foreign object due to esophageal contraction, deep embedding, bleeding, or other circumstances, as well as potential complications such as mucosal damage, bleeding, perforation, and infection (10).

### 2.5. Statistical analysis

The statistical analysis was conducted using SPSS 23.0 software. Normally distributed measurement data were presented as X ± s, and intergroup comparisons were performed using a *t*-test. Skewed measurement data were expressed as M (Q1, Q3), and the non-parametric Mann–Whitney method was used for intergroup comparisons. Numerical data were reported as the number of cases, and the chi-squared test or Fisher's exact probability method was used for intergroup comparisons. A significance level of *P* <0.05 was utilized to determine statistical significance.

## 3. Results

The study included a total of 60 cases, with each group consisting of 30 cases. Table 1 lists the types of foreign objects, while Table 2 displays baseline characteristics of the two groups.

**Table 1:** Foreign body types of the two groups.

Group	Foreign body types				
	Fish bone	Other bone tissue	Denture	Jujube pit	Other hard foreign body
Self-developed sleeve	11	8	3	5	3
Transparent cap	16	9	2	2	1

**Table 2:** Baseline characteristics of the two groups.

Characteristic	Self-developed sleeve	Transparent cap	*P*-value
	*n* = 30	*n* = 30	
Age, years [M (Q1, Q3)]	59 (45, 67)	55 (45, 62)	0.147
Gender, *n* (%)			0.36
Male	5 (17)	8 (27)	
Female	25 (83)	22 (73)	
Length, mm [M (Q1, Q3)]	25 (20, 30)	254 (20, 25)	0.066
Width, mm [M (Q1, Q3)]	8 (3, 12)	9 (6, 12)	0.242
Location of foreign body, *n* (%)			
Cervical esophagus	23 (77)	24 (80)	
Thoracic esophagus	7 (23)	6 (20)	
Esophagus abdominal segment	0 (0)	0 (0)	

Compared to the transparent cap, the self-developed sleeve method effectively safeguards the mucosa at the entrance of the esophagus, reducing further mucosal damage after the removal of the foreign object. This not only ensures a clearer visual field but also shortens the operation duration and lessens the prevalence of intra- and postoperative complications during the removal of embedded foreign objects (Table 3). The self-developed sleeve method demonstrated a success rate of 100%, while that of the transparent cap method was 94%.

**Table 3:** Comparison of outcomes between the two groups.

Outcome measure	Self-developed sleeve	Transparent cap	*P*-value
	*n* = 30	*n* = 30	
Duration, s [M (Q1, Q3)]	40 (10, 50)	80 (10, 90)	0.001
Success, *n* (%)			0.529
Yes	30 (100)	28 (93)	
No	0 (0)	2 (7)	
Additional mucosal tear at the entrance of the esophagus, mm [M (Q1, Q3)]	0 (0, 0)	4.0 (0, 6)	<0.001
Additional mucosal tear at the impacted site, mm [M (Q1, Q3)]	0 (0, 2)	6.0 (3, 8)	<0.001
Clearness, *n* (%)			<0.001
A	21 (70)	4 (13)	
B	6 (20)	6 (20)	
C	3 (10)	20 (67)	
Adverse events, *n* (%)			
Perforation	0	0	
Mucosal bleeding	7 (23)	28 (67)	0.001
Infection	0	0	

**Figure 2:**
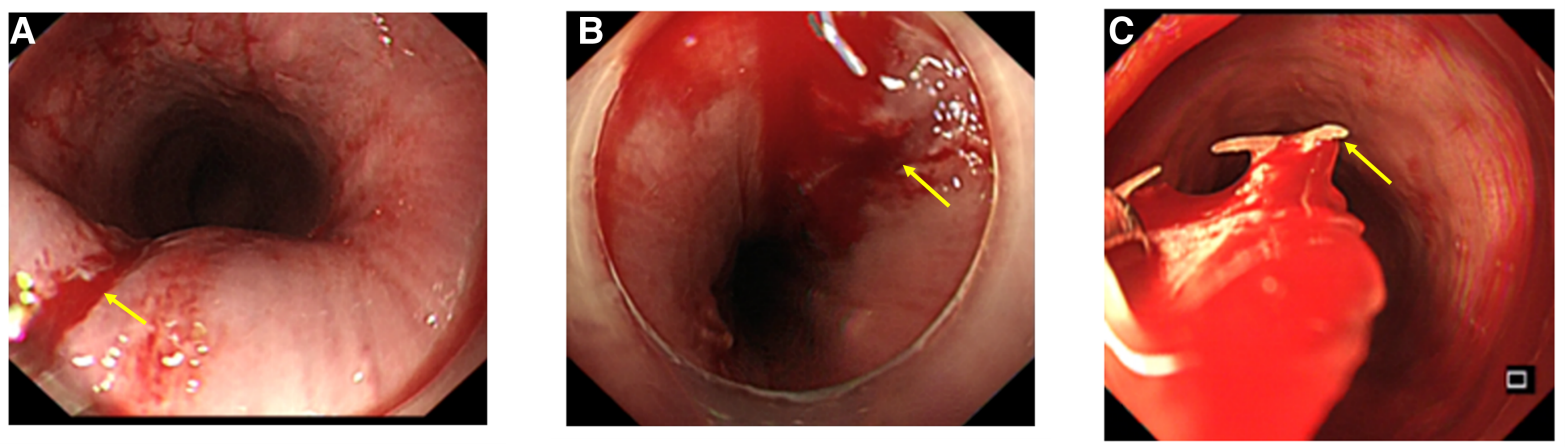
When foreign objects are extracted from the conventional clear cap, an injury to the mucosal lining of the esophagus may occur, including scratching and bleeding. When foreign objects larger than the diameter of the clear cap are extracted, as shown in (**A**), it may cause scratching and bleeding at the esophageal inlet as indicated by the yellow arrow. In (**B**), the impaction of larger foreign bodies may not be resolved during removal, leading to scratching and bleeding of the impacted site due to the abrupt pulling of the foreign object. In addition, (**C**) depicts how the irregular removal of foreign objects can lead to scratching and bleeding.

Removing foreign objects using the transparent cap group can be arduous and can lead to forceful extraction, thereby causing new injuries at the site of incarceration and at the entrance of the esophagus (Figure 2). Therefore, an independent development of a foreign object removal sleeve was created (Figure 1). The endoscope's distance from the sleeve tip is around 0.4 cm (0.2 cm invisible + 0.2 cm visible). Using the pressure applied to the endoscope's end, the incarceration is directly removed, causing the tip of the foreign object to enter and be removed through the sleeve or pulled into the sleeve using a foreign object clamp. The incarceration site is free from new damage, and the sleeve is delicately removed from the body, protecting the food inlet from scratches. In addition, the sleeve allows for compression hemostasis and a clear observation of the depth of the injury through transparent tubes.

**Figure 3:**
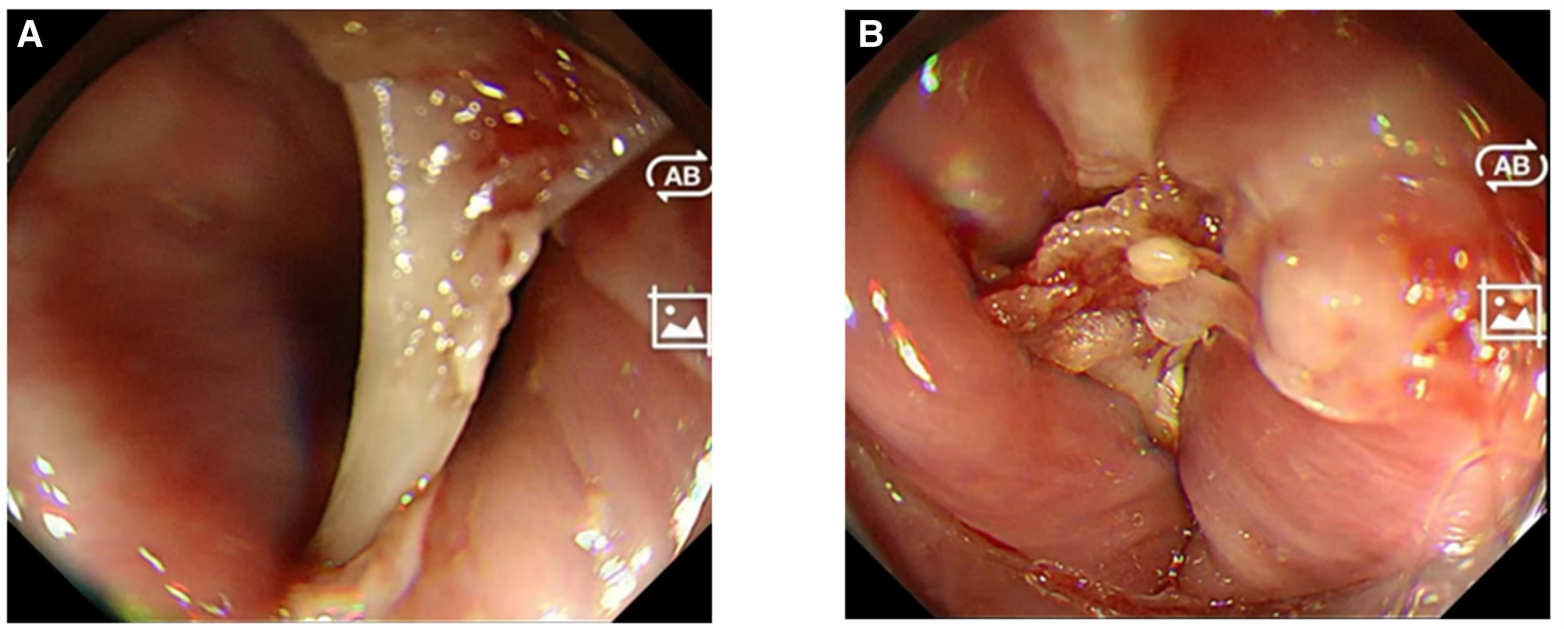
The transparent cap group revealed that (**A**) one end of the foreign body was wedged into the esophageal wall and could not be released and extracted by the clear cap; consequently, it must be forcibly removed, leading to a significant and unavoidable additional mucosal damage. Similarly, in (**B**), the depth of impaction of the foreign body on the other side remains obscured, precluding its release and extraction.

**Figure 4:**
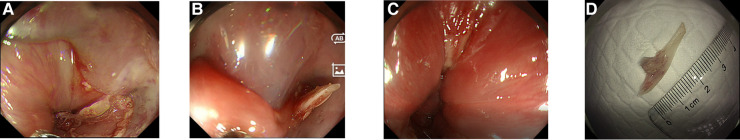
The same foreign body seen in Figure 3, which was removed using the self-developed sleeve. The sheath was used to press on one end of the foreign body, releasing it from entrapment as shown in (**A**). The sharp tip of the foreign body was then inserted into the self-developed sleeve for a controlled removal as displayed in (**B**), preventing additional injury to the surrounding structures. Following foreign body extraction, no new wounds were identified in the area as demonstrated in (**C**). Finally, the successful removal of the foreign body was confirmed as shown in (**D**).

The visual capacity of the transparent cap group is inadequate, making it challenging to visualize the incarcerated tip and remove it entirely. The manipulation process often damages the incarceration further. In addition, if the width of the foreign object exceeds the protective scope of the transparent cap, it can scratch the narrow entrance, subsequently causing additional damage (Figure 2). One participant assigned to the transparent cap group required an alternative method and was provided with a self-developed sleeve. After conducting a meticulous evaluation, we found that the patient depicted in Figure 3 was not a suitable candidate for using a transparent cap to remove foreign objects. Thus, during the surgical procedure, we employed a self-developed sleeve as an alternative method (Figure 4).

Figure 4 depicts the same foreign object as in Figure 3, with the sleeve's tip pressing against the incarceration while delicately extracting it, allowing the tip of the foreign object to move smoothly into the sleeve, where it is then carefully extracted with the long axis of the foreign body parallel to it that effectively protects it. The incarceration site remains free from new damages throughout the entire process. We have recorded a video to dynamically demonstrate the excellent functionality of the self-developed sleeve in safely and effectively extracting foreign objects. This video is included as Supplementary Material S1.

## 4. Discussion

Typically, about 80%–90% of foreign bodies in the UGIT are naturally expelled through digestion, while 10%–20% require removal under endoscopic guidance, with only 1% necessitating surgical intervention or involving complications. When foreign objects have sharp edges, emergency endoscopic surgery is recommended within 2–6 h (11). Refractory foreign objects are particularly difficult to diagnose and treat under emergency endoscopy. A detailed medical history helps guide the appropriate imaging modalities, and the urgency of therapeutic intervention is established accordingly (12, 13).

The overtube is a commonly used auxiliary device in endoscopy for removing sharp foreign bodies within the upper digestive tract (9). According to existing literature, there are various overtube types with an inner diameter of 14–21 mm and a length range of 23–135 cm (14). Nevertheless, clinical practice often reveals that foreign bodies are too large to be pulled into the overtube due to their limited inner diameter (15). In terms of incarceration removal, innovative external sleeve solutions have been suggested by some studies (16, 17), which propose the use of a balloon to expand the esophageal wall and loosen foreign objects.

However, when the foreign object is large, or its sharp edges get deeply embedded within the esophageal wall, balloon dilation may be ineffective in removing the incarceration and may harm surrounding organs in the process. In addition, balloon dilation cannot safeguard the esophageal entrance if the foreign object is incarcerated at the entrance, and the space available for balloon diastolic operation is often limited.

Our self-developed sleeve has an inner diameter of 18 mm and a slope length of 25 mm, similar to the diameter of the esophagus. Made of soft materials, the sleeve fits comfortably with the endoscope, therefore, causing no discomfort. It can remove foreign objects with a transverse measurement no larger than 2.5 cm (not accounting for length), encompassing nearly all foreign bodies that may embed within the esophagus.

The distal part of the sleeve is inclined, a design that enhances the clearance of sharp foreign objects partially embedded in the esophageal wall by pressing against the wall, unlike a flat distal end. In this study, two patients from the transparent cap group who had irretrievable deeply embedded foreign objects were successfully treated with the innovative sleeve.

In contrast to the transparent cap employed in this study, the self-developed sleeve offers advantages. It removes embedded foreign objects by applying pressure to the esophageal wall in order to prevent secondary damage caused by the dislodging of mucosa at the embedment.

As a result, the entrance to the esophagus remains intact, undergoing no tearing during the process of the foreign object removal. After the extraction of the foreign object, the external pressure of the outer sleeve is instrumental in preventing mucosal bleeding and ensuring a clear visual field for inspecting the wound's depth, thereby highlighting the safety advantages of the self-developed sleeves. Despite the similarities in terms of the success rate in foreign object removal in this study, differences could not be significantly established among the three tools because of the sample size and the expertise of the endoscopist.

The primary limitations of this study are as follows: first, it is a single-center study with a small sample size, and the validity of our findings remains to be established through multisite studies. Second, despite stringent quality control measures, uncontrollable confounding variables may have impacted the results of our experimental study.

In summary, we present a self-developed sleeve endoscopic-assisted device for foreign body removal in this study. This device is handy in the management of stubborn sharp foreign bodies embedded in the UGIT. The application of this self-developed sleeve reduces operative time. It minimizes mucosal damage during object embedding, protecting the entrance to the esophagus, thereby maintaining a clearer visual field and avoiding postoperative mucosal bleeding. This device offers both efficacy and safety advantages and holds significant potential for clinical use and dissemination.

## Data Availability

The original contributions presented in the study are included in the article/Supplementary Material, further inquiries can be directed to the corresponding authors.
